# Evaluation of host genetics on outcome of tuberculosis infection due to differences in killer immunoglobulin-like receptor gene frequencies and haplotypes

**DOI:** 10.1186/s12863-015-0224-x

**Published:** 2015-06-16

**Authors:** Kali Braun, Joyce Wolfe, Sandra Kiazyk, Meenu Kaushal Sharma

**Affiliations:** Department of Medical Microbiology, University of Manitoba, 543-745 Bannatyne Avenue, Winnipeg, R3E 0 J9 MB Canada; National Reference Centre for Mycobacteriology, Public Health Agency of Canada, 1015 Arlington Street, Winnipeg, R3E 3R2 MB Canada

**Keywords:** Killer immunoglobulin-like receptor, KIR profiles, KIR haplotypes, Tuberculosis

## Abstract

**Background:**

Outcome of *Mycobacterium tuberculosis* (Mtb) infection is affected by virulence of the infecting strain of Mtb*,* host environment, co-morbidities, and the genetic composition of the host, specifically the presence or absence of genes involved in immune responses/regulation. It is hypothesized that specific killer immunoglobulin-like receptor (KIR) genes may be associated with Mtb infection and clinical outcome. This cross-sectional study examined the KIR gene frequencies, profiles, and haplotypes of individuals with active tuberculosis, latent tuberculosis infection, compared to TB and HIV negative healthy controls.

**Results:**

Analysis of KIR gene frequencies revealed differences among disease status groups, suggesting that enrichment or depletion of specific KIR genes may direct the disease outcome. Mtb infected individuals were more likely to have a centromeric-AA haplotype compared to controls.

**Conclusion:**

The differences in KIR gene frequencies and haplotypes may result in differential cytokine expression, contributing to different disease outcomes, and suggest a genetic influence on Mtb susceptibility and pathogenesis.

## Background

Tuberculosis (TB) incidence in the world and in Canada overall are declining toward goals set by the World Health Organization [1], however, certain populations and/or regions within Canada continue to have rates of tuberculosis exceeding the national average. In 2012, the global burden of TB was estimated at 8.6 million incident cases (122/100,000 population) [1]. Canada reported 1686 new active cases of TB (4.8/100,000) in 2012 [2]. The incidence of TB in Manitoba was more than twice the national rate at 138 cases (10.9/100,000).

The immune response to an intracellular pathogen such as *Mycobacterium tuberculosis* (Mtb) involves natural killer (NK) cells to bridge the innate and adaptive immune response to infection [3]. NK cells are important in early Mtb infection, as they are capable of activating phagocytic cells at the site of infection [4], and are producers of INF-γ, which functions to activate macrophages [5]. The activity of NK cells is controlled by a balance of inhibitory and stimulatory signals generated when human leukocyte antigen (HLA) class I ligands bind to killer immunoglobulin-like receptors (KIRs) on the NK cell surface [6]. This highly specific recognition system is controlled by the integration of signals generated by a multitude of inhibitory and activating KIRs, which inhibit or activate, respectively, cytotoxicity and secretion of cytokines ultimately leading to death of the targeted cell [7]. Both inhibition and activation involve a number of signalling molecules, as previously described [8, 9].

There is extensive genomic diversity in KIR genes in humans. Currently, a database and online repository for immune gene frequencies in worldwide populations reports 517 different KIR genotypes [10, 11]. It is believed that this variation may affect resistance or susceptibility to a number of pathogens through ligand-receptor interactions and the downstream signalling and/or cytokine release that follows [12, 13]. Genetic susceptibility or resistance to infectious diseases, in conjunction with environmental and host risk factors, is thought to determine disease progression [14–16].

Present literature indicates that the outcome of Mtb infection is affected not only by virulence of the infecting strain of *M. tuberculosis* [17], but also by host environment, disease co-morbidities, and the genetic composition of the host, specifically the presence or absence of genes that regulate the immune system [14, 16, 18–20]. Following Mtb infection, approximately 10 % of individuals will develop active TB (ATB) during their lifetime, while the majority of individuals will exhibit latent TB infection (LTBI) [21, 22]. LTBI refers to the condition in which Mtb remains viable in the macrophage but retains a small amount of metabolic activity [23]. It is not currently known which genes and/or immune components regulate an individual’s disease outcome following exposure (ATB, LTBI, or exposed uninfected). Present literature captures only those studies focusing on genetic profiles among active tuberculosis vs. uninfected individuals. In the majority of these studies, the control group contains both individuals with LTBI infection as identified by a positive Tuberculin skin test (TST), and those with uninfected status.

The novel aspect of this study is to identify unique profiles among the LTBI population, diagnosed using the Interferon gamma release assay (IGRA)' as there is identify twice in this sentence. Differences in KIR profiles and haplotypes may be associated with Mtb infection status [24–26] and play a role in altered TB disease progression and disease outcomes. In this cross-sectional study, we examined the enrichment or depletion of KIR genes in individuals from Manitoba with ATB infection, LTBI and controls, and further explored the association between Mtb infection status and KIR profiles and haplotypes.

## Methods

### Sample Populations

The 209 samples consisted of whole blood from individuals living in Manitoba. The sampling was performed at hospital and community TB clinics in Winnipeg, Manitoba, Canada between November 3, 2009 and March 29, 2011 and was cross-sectional in nature. The study was approved by the Health Research Ethics Board at the University of Manitoba (H2008:301). All study participants provided written informed consent following consultation with a study nurse. ATB infection (*n* = 59) was confirmed by mycobacterial culture. LTBI (*n* = 46) was identified using the interferon-gamma release assay (IGRA) (QuantiFERON®-TB-Gold, Qiagen). Healthy IGRA negative HIV negative individuals (*n* = 104) were used as controls and consisted largely of individuals participating in routine occupational health screening, and immigration screening. All individuals within the specified time period who consented to genetic testing were included in this study. Exclusion criteria included those individuals with HIV co-infection, and anyone who exhibited an indeterminate IGRA response. Participant demographics for ethnicity, age, and gender can be seen in Table 1.

**Table 1:** Sample demographics

Parameter	Value	Number	Percent
Gender	Male	91	43.5
	Female	118	56.5
Age	≤19	0	0
	20 - 39	88	42.1
	40 - 59	105	50.2
	≥60	16	7.7
Disease status	Control	104	49.8
	LTBI	46	22
	ATB	59	28.2
Ethnicity	Canadian-born	131	62.7
	Control	72	54.9
	LTBI	12	9.2
	ATB	47	35.9
	Foreign-born	78	37.3
	Control	32	41.0
	LTBI	34	43.6
	ATB	12	15.4

*LTBI* Latent tuberculosis infection; *ATB* Active tuberculosis

### DNA extraction and replication

Genomic DNA was extracted using Qiagen DNA Mini Kit as per manufacturer’s instructions (Qiagen, Louisville, KY). The samples were subjected to whole genome replication using the Qiagen Repli-G mini kit as per manufacturer’s instructions to increase DNA concentration of the testing sample.

### KIR genotyping

The concentration of DNA was normalized to 100 μg/mL at 260 nm using the SmartSpec Plus spectrophotometer (Bio-Rad, Mississauga, ON). KIR genotyping was performed by sequence-specific primer polymerase chain reaction using the Miltenyi Biotec KIR Typing Kit (Auburn, CA) as previously described [9]. The amplicons were visualized with UV light (Bio-Rad Gel Doc EZ Imager, Mississauga, ON) following gel electrophoresis at 13 V/cm on a 2 % agarose gel containing ethidium bromide. The KIR typing kit allows for detection of all known human KIR genes and alleles [27, 28]. KIR2DL5A and KIR2DL5B are collectively referred to as KIR2DL5 for this paper.

### Statistical analysis

Data for each individual was entered into BioNumerics software version 5.0 (Applied Maths, Belgium) as binary character data. All KIR genes were combined into a single KIR profile for each individual and clustered to identify prevalent profiles among specified groups using the categorical co-efficient and unweighted pair group method with arithmetic mean (UPGMA) [29]. KIR gene frequencies were tabulated by direct counts from the clustered profiles to determine frequency within a defined group. Differences between Mtb infection status groups were estimated using the two-tailed Fisher’s exact test (GraphPad Software, La Jolla, CA). A *P*-value ≤0.05 was considered statistically significant. Haplotype designation was determined as previously described [9].

## Results

### KIR gene frequencies

In order to determine the differences in KIR gene frequencies between different disease status groups (ATB, LTBI, and controls), the KIR gene frequency data obtained was analyzed and compared. All 209 samples consistently contained the framework genes KIR2DL4, KIR3DL2, KIR3DL3, and the pseudogenes KIR2DP1 and KIR3DP1.

Five KIR genes (KIR2DL2, KIR2DL5, KIR2DL5B, KIR2DS2, and KIR2DS3) differed significantly (*P* ≤ 0.05) in frequency between disease status groups (Table 2). Two genes differed between individuals with Mtb infection (LTBI and ATB) vs. controls, KIR2DL2 (33.33 % vs. 55.77 %, *P* = 0.0014) and KIR2DS2 (34.29 % vs. 54.81 %, *P* = 0.0035). However, the underlying differences can be exposed when analyzing LTBI and ATB separately. KIR2DL5 and KIR2DL5B (both 73.91 % vs. 51.92 %, *P* = 0.0125) were present in higher frequency in individuals within the LTBI group as compared to controls. KIR2DL2 (27.12 % vs. 55.77 %, *P* = 0.0005), KIR2DS2 (27.12 % vs. 54.82 %, *P* = 0.0010), and KIR2DS3 (8.47 % vs. 30.77 %, *P* = 0.0009) were present in a lower frequency in individuals with ATB compared to controls. Lastly, gene frequencies of KIR2DL5 (73.91 % vs. 49.15 %, *P* = 0.0156), KIR2DL5B (73.91 % vs. 49.15 %, *P* = 0.0156), and KIR2DS3 (39.13 % vs. 8.47 %, *P* = 0.0002) differed significantly between latently and actively infected individuals, respectively.

**Table 2:** Killer immunoglobulin-like receptor (KIR) gene frequencies by tuberculosis status

	KIR; n (% f)																		
	2DL1	2DL2	2DL3	2DL4	2DL5all	2DL5A	2DL5B	2DS1	2DS2	2DS3	1D	2DS4	2DS5	3DL1	3DL2	3DL3	3DS1	2DP1	3DP1
Mtb Infected																			
All (*n* = 105)	103 (98.10)	35 (33.33)	100 (95.24)	105 (100.00)	63 (60.00)	48 (45.71)	63 (60.00)	50 (47.62)	36 (34.29)	23 (21.90)	73 (69.52)	105 (100.00)	41 (39.05)	99 (94.29)	105 (100.00)	105 (100.00)	55 (52.38)	105 (100.00)	105 (100.00)
LTBI (*n* = 46)	45 (97.82)	19 (41.30)	42 (91.30)	46 (100.00)	34 (73.91)	24 (52.17)	34 (73.91)	25 (54.35)	20 (43.48)	18 (39.13)	36 (78.26)	46 (100.00)	16 (34.78)	45 (97.82)	46 (100.00)	46 (100.00)	26 (56.52)	46 (100.00)	46 (100.00)
ATB (*n* = 59)	58 (98.31)	16 (27.12)	58 (98.31)	59 (100.00)	29 (49.15)	24 (40.68)	29 (49.15)	25 (42.37)	16 (27.12)	5 (8.47)	37 (62.71)	59 (100.00)	25 (42.37)	54 (91.53)	59 (100.00)	59 (100.00)	29 (49.15)	59 (100.00)	59 (100.00)
Control (*n* = 104)	102 (98.08)	58 (55.77)	95 (91.35)	104 (100.00)	54 (51.92)	43 (41.35)	54 (51.92)	44 (42.31)	57 (54.81)	32 (30.77)	79 (75.96)	103 (99.04)	32 (30.77)	97 (93.27)	104 (100.00)	104 (100.00)	43 (41.35)	104 (100.00)	104 (100.00)
*P*-value																			
Mtb Infected vs. Control	1.0000	**0.0014**	0.2837	1.0000	0.2664	0.5776	0.2664	0.4879	**0.0035**	0.1599	0.3519	0.4976	0.2463	0.7832	1.0000	1.0000	0.1279	1.0000	1.0000
LTBI vs. Control	1.0000	0.1137	1.0000	1.0000	**0.0125**	0.2853	**0.0125**	0.2141	0.2190	0.3505	0.8363	1.0000	0.7050	0.4357	1.0000	1.0000	0.1099	1.0000	1.0000
ATB vs. Control	1.0000	**0.0005**	0.0957	1.0000	0.7472	1.0000	0.7472	1.0000	**0.0010**	**0.0009**	0.1047	1.0000	0.1716	0.7583	1.0000	1.0000	0.4121	1.0000	1.0000
ATB vs. LTBI	1.0000	0.1474	1.0000	1.0000	**0.0156**	0.3237	**0.0156**	0.2432	0.0988	**0.0002**	0.0933	1.0000	0.5457	0.2272	1.0000	1.0000	0.5552	1.0000	1.0000

Significant P-values (≤0.05) are bolded; *Mtb* Mycobacterium tuberculosis, *LTBI* Latent tuberculosis infection, *ATB* Active tuberculosis

### KIR gene profiles

**Fig. 1:**
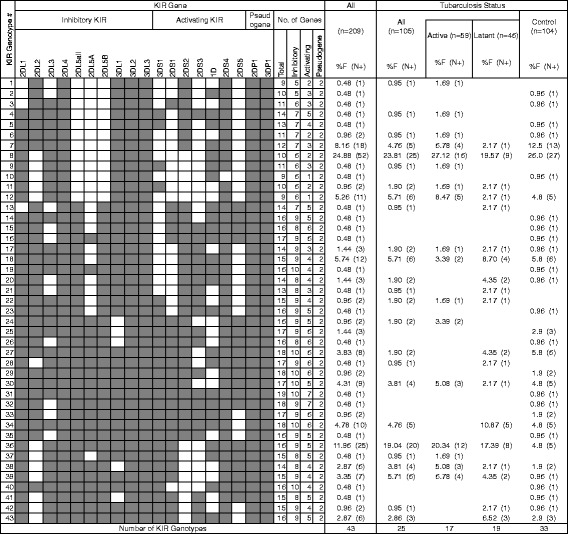
Frequency of KIR genotypes in study group population. Forty-three distinct KIR types were seen in these 209 individuals that differ from each other by the presence of (shaded box) or absence (white box) of 19 KIR genes (KIR2DL5 broken down into 2DL5A, 2DL5B, and 2DL5 (both A and B); KIR2DS4 broken down into 1D and full length 2DS4). Frequency (%F) of each genotype is expressed as a percentage and is defined as the number of individuals having the genotype (N+) divided by the number of individuals (n) in the tuberculosis status group

**Fig. 2:**
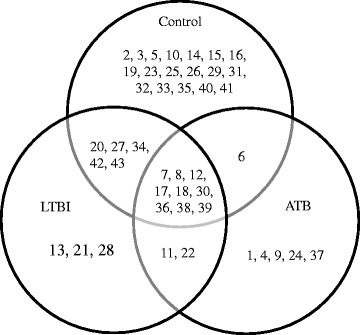
Distribution of KIR genotypes among active tuberculosis (ATB), latent tuberculosis infection (LTBI), and controls. Content of each genotype can be seen in Fig. 1

Forty-three KIR profiles (genotypes) were identified in this study (Fig. 1). These profiles ranged in their frequency of distribution from as high as 24.88 % (52/209) to as low as 0.48 % (1/209). Twenty-two of the 43 profiles identified were unique to a single individual. The most prominent genotypes were #7, (18/209, 8.2 %), #8 (52/209, 24.9 %), #12 (11/209, 5.3 %), #18 (12/209, 5.7 %), and #36 (25/209, 12.0 %). Eight KIR genotypes were shared between all three disease status groups (ATB, LTBI, controls; genotypes # 7, 8, 12, 17, 18, 30, 36, 39) (Fig. 2). Excluding those shared with individuals from the control group, two genotypes were shared between the LTBI and ATB groups (genotypes #11, 22). Nineteen genotypes were exclusive to the control group. Five genotypes were exclusive to those individuals with ATB (genotypes #1, 4 9, 23, 37), and represented 6/59 (10.2 %) active cases. Three genotypes were exclusive to those individuals with LTBI (genotypes # 13, 21, 28), and represented 3/46 (6.5 %) latent cases.

Those individuals with Mtb infection (LTBI or ATB) were found within 25 of the 43 genotypes, most predominantly in #8 (25/105, 23.8 %) and #36 (20/105, 19.0 %). Over 42 % of Mtb infected individuals were found in these two genotypes.

### Haplotype analysis

In addition to gene frequency variation, there is haplotypic variation due to the different number and kinds of KIR genes [30]. Both LTBI (34.8 %, *P* = 0.0004) and ATB (32.3 %, *P =* 0.0005) infected individuals were significantly more likely to have AA-AB haplotypes than controls (9.6 %; Table 3). Additionally, those individuals with ATB were less likely to have an AB-AB haplotype compared to controls (8.5 % vs. 21.2 %, *P* = 0.0476). The majority of the Mtb infected cases are represented in the AA-AA (LTBI – 21.7 %, ATB – 35.6 %) and AA-AB (LTBI – 34.8 %, ATB – 32.3 %) haplotypes. Overall, 66.67 % of Mtb infected individuals had a centromeric-AA haplotype (LTBI – 58.7 %, ATB – 72.9 %), compared with only 44.2 % of controls (*P* = 0.0014).

**Table 3:** Frequency of centromeric and telomeric haplotypes by tuberculosis status

Haplotype		Tuberculosis Status; *n* (%F)		
		Latent	Active	Control
Centromeric	Telomeric	(*n* = 46)	(*n* = 59)	(*n* = 104)
AA	AA	10 (21.7)	21 (35.6)	33 (31.73)
AA	AB	16 (34.8)	19 (32.3)	10 (9.62)^a,b^
AB	AA	6 (13.0)	8 (13.6)	24 (23.08)
AB	AB	9 (19.6)	5 (8.5)	22 (21.15)^b^
AA	BB	1 (2.2)	3 (5.1)	3 (2.88)
AB	BB		2 (3.4)	3 (2.88)
BB	AA	4 (8.7)	1 (1.7)	4 (3.85)
BB	AB			4 (3.85)
BB	BB			1 (0.96)

^a^Significant compared latent TB; ^b^Significant compared to active TB; *p*-value ≤ 0.05 considered significant

## Discussion

This study was designed to investigate the KIR gene frequencies in individuals from Manitoba with ATB, LTBI, and a control group, as described in methods. Additionally, KIR profiles and haplotypes were analyzed. KIR genes may influence disease outcome (latent vs. active) which is controlled in part by an organized immune response.

When determining KIR gene frequencies, framework genes and pseudogenes were present in 100 % of the samples, as expected [31]. KIR2DL2, KIR2DL5, KIR2DL5B, KIR2DS2, and KIR2DS3 differed significantly between Mtb status groups.

Mahfouz et al. and Mendez et al. both found KIR2DL3 to be the only statistically significant KIR gene frequency to differ between ATB patients and controls (higher in ATB patients; *P* = 0.03 and *P* = 0.02, respectively) [24, 25]. In our study, KIR2DL3 occurred in only a slightly higher frequency of individuals with ATB (98.31 %) compared to controls (91.35 %, *P* = 0.0957; NS). Pydi et al. found gene frequencies of KIR2DS1, KIR2DS5, KIR3DL1, and KIR2DL3 to be higher in ATB patients compared to controls (*P* < 0.0001) [32]. Although KIR2DS5 and KIR2DL3 gene frequencies differed slightly between ATB and control groups in our study, none of the observed differences were statistically significant. Lastly, Lu et al. found gene frequencies of KIR2DS1, KIR2DS3, and KIR3DS1 to be significantly higher in ATB patients compared to controls (*P* < 0.05) [33]. In our study, frequencies of KIR2DS1 and KIR3DS1 were not statistically different between those with ATB compared to controls. However, when looking at KIR2DS3, our study showed a decreased frequency in individuals with ATB compared controls (8.47 % vs. 30.77 %, *P* = 0.0009). Observed differences in KIR frequencies between TB status groups may be due in large part to ethnicity, as in each of the above mentioned studies, a different country of origin was involved, and KIR are known to differ among ethnic groups [11]. Given that the Manitoba population is very heterogeneous consisting of many foreign born immigrant individuals, as well as indigenous and Canadian born populations, future studies matching TB status groups by ethnicity will help to more clearly define the role of KIR genes in TB pathogenicity. Additionally, group definitions may have played a role; the control groups in the above published studies contained either TST positive individuals or had no data on TB reactivity. These control group then reflect a mixture of both uninfected and LTBI infected participants. In contrast to this, with the use of the IGRA testing, we were able to clearly distinguish our LTBI and control groups.

With the exception of KIR2DL2, our study found an increased presence of inhibitory KIR (KIR2DL5, KIR2DL5B) in LTBI individuals and a decreased presence of activating KIR (KIR2DS2, KIR2DS3) in ATB infected individuals. This may suggest that the enrichment or depletion of specific KIR genes predisposes an individual to progressing to ATB disease by means of an inadequate cytotoxic response to the pathogen. LTBI refers to the condition in which Mtb remains viable in the macrophage but only retains a small amount of metabolic activity [23]. The inability of the immune system to maintain the infection in a latent state results in ATB infection.

Forty-three different gene profiles were identified in the 209 samples, of which, 25 profiles contained Mtb cases. The profiles containing the most Mtb cases were also prevalent in the control group, suggesting an unlikely correlation between profile/genotype and TB status. Many profiles were unique to individuals with Mtb, however there is little to be concluded from those profiles containing only a few individuals. Extrapolation of these findings via continued sampling is warranted to determine the importance of KIR profiles.

Those individuals with LTBI and ATB were more likely to have an AA-AB haplotype than controls. This was the haplotype that contained the most Mtb cases (35/105, 33.3 %). Two-thirds (66.7 %) of individuals with TB had a centromeric-AA halpotype, compared to only 44.2 % of controls (*P* = 0.0014). A centromeric-AA haplotype represents the haplotype with the fewest number of activating genes. It is hypothesized that this lack of activating genes may prevent the appropriate release of *M. tuberculosis* killing cytokines [8].

A limitation to this study is the lack of longitudinal data among our LTBI status group. As we do not know when an individual became LTBI (IGRA positive) it is possible that some of these individuals went on to develop primary or secondary TB, however we do not have access to this data. It is known that these were healthy individuals with low risk for the development of ATB. Another limitation is the unknown TB exposure of our control group (IGRA negative), we can make no conclusions in regards to the role KIR plays in TB susceptibility. This group is used as a reference comparison group for our LTBI and ATB populations.

## Conclusions

In summary, major differences can be seen in KIR gene frequencies across Mtb disease status groups. KIR haplotype frequencies differ between these groups as well. The differences in KIR gene frequencies and/or haplotypes may result in differential cytokine expression, contributing to different disease outcomes, and suggest a genetic influence on Mtb susceptibility and pathogenesis. The skewed distribution of A-containing centromeric haplotypes (containing fewer activating genes), along with the increased presence of TB disease in these haplotypes, suggests a correlation. Further investigation is needed to characterize the subtleties of these differences by way of sequencing of specific KIR genes and/or KIR-HLA association studies taking into account different ethnic populations.

### Availability of supporting data

The data set supporting the results of this article is included within the article.

## Abbreviations

Mtb: *Mycobacterium tuberculosis*
KIR: Killer immunoglobulin-like receptor
TB: Tuberculosis
NK: Natural killer
HLA: Human leukocyte antigen
ATB: Active tuberculosis
LTBI: Latent tuberculosis infection
TST: Tuberculin skin test
UPGMA: Unweighted pair group method with arithmetic mean
